# [Expression of Concern] Pifithrin-μ is efficacious against non-small cell lung cancer via inhibition of heat shock protein 70

**DOI:** 10.3892/or.2026.9123

**Published:** 2026-04-22

**Authors:** Yang Zhou, Jingping Ma, Jiahong Zhang, Li He, Jianhua Gong, Cong Long

Oncol Rep 37: 313–322, 2017; DOI: 10.3892/or.2016.5286

Following the publication of the above paper, it was drawn to the Editor's attention by a concerned reader that Fig. 6A on p. 320, showing the tumor volumes from the *in vivo* experiments, was lacking the units in the y-axis, hence undermining the ability to usefully interpret the data in this figure part. In addition, after having performed an independent assessment of the data in this paper in the Editorial Office, it came to light that western blot data in Fig. 2D and flow cytometric plots in Fig. 3A appeared subsequently in different papers in different experimental contexts, one written by different authors in 2018 in the journal *Journal of Experimental and Clinical Cancer Research*, and the other featuring an author named Cong Long in the journal *Oncology Letters*.

The authors have been contacted by the Editorial Office to offer an explanation for the concerns that have been identified in their paper, and we are awaiting their response. Owing to the fact that the Editorial Office has been made aware of potential issues surrounding the scientific integrity of this paper, we are issuing an Expression of Concern to notify readers of these potential problems while the Editorial Office continues to investigate this matter further.

